# Micro-nanoscale laser subsurface vertical modification of 4H-SiC semiconductor materials: mechanisms, processes, and challenges

**DOI:** 10.1186/s11671-025-04309-4

**Published:** 2025-07-16

**Authors:** Hongmei Li, Hongwei Wang, Yuxin Li, Xiwen Lu, Lin Li, Yinzhou Yan, Wei Guo

**Affiliations:** 1 Ningbo Institute of Materials Technology and Engineering, Chinese Academy of Sciences, Ningbo, 315201 China; 2 School of Physics and Optoelectronic Engineering, Beijing University of Technology, Beijing, 100124 China

**Keywords:** Silicon carbide semiconductor, Laser modification, Wafer stripping, Nonlinear absorption, Laser pulse control

## Abstract

Wide-bandgap semiconductor materials, exemplified by silicon carbide (SiC), have emerged as pivotal materials in semiconductor devices due to their exceptional chemical stability, high electron mobility, and thermal stability. With the rapid development of microelectronic devices and integrated optical circuits, the demand for high-yield and high-quality processing of SiC wafer has intensified. Traditional SiC wafer processing technologies suffer from low efficiency and high material loss, making it difficult to meet industrial demands. Therefore, the development of efficient, low-damage processing techniques has become a pressing issue in the SiC wafer processing field. Ultrashort pulsed laser processing, with its advantages of contact free processing, no mechanical stress, and small heat-affected zones, has garnered significant attention in SiC wafer processing in recent years. By generating a modified layer within the material, laser processing plays a crucial role in wafer fabrication. However, the key challenge lies in precisely controlling the thickness of the modified layer down to the micro-nano scale to minimize material loss. This review systematically discusses the interaction mechanisms and modification processes of laser with wide-bandgap semiconductor SiC materials. It focuses on the core issue in laser modification technology, where nonlinear effects make it difficult to precisely control the modification layer depth, thereby affecting both modification quality and processing efficiency. To address this, the paper summarizes the differences in modification mechanisms with lasers of varying pulse durations and proposes a multi-strategy solution to improve modification quality and processing efficiency through pulse control and synergistic optimization of process parameters. Additionally, this review provides a comprehensive overview of advanced SiC wafer detachment processes, including cold cracking stripping, chemically assisted stripping, ultrasonic stripping, and multi-laser composite stripping, and identifies the primary challenges and future directions in the field of SiC wafer processing.

## Introduction

**Fig. 1:**
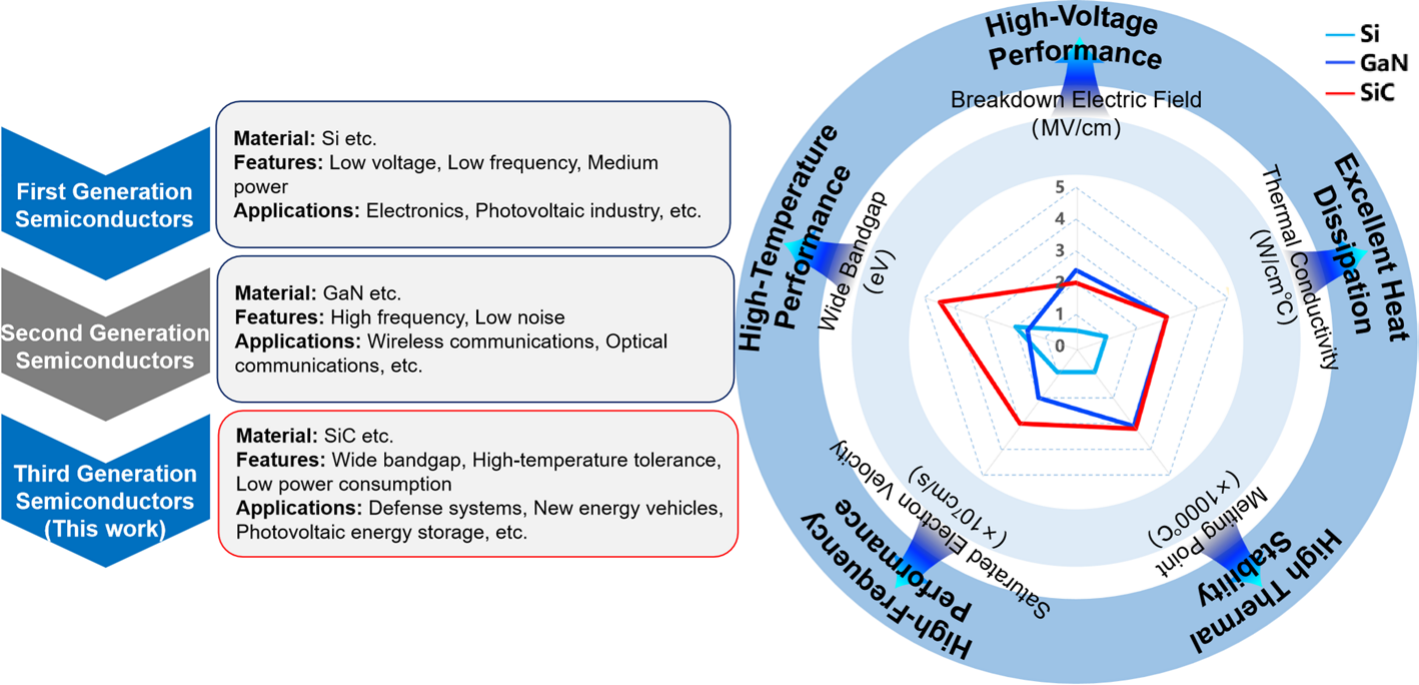
Features and applications of first, second, and third-generation semiconductor materials, along with a performance comparison of Si, GaN, and SiC. (Adapted from reference [23])

With the rapid development of fields such as electronics, aerospace, and new energy [1, 2], the demand for high-performance wide-bandgap semiconductor materials is growing. SiC, as the third-generation semiconductor material following silicon (Si) and gallium arsenide (GaAs) [3–8], is an ideal material for high-temperature, high-frequency, and complex radiation environments. Taking 4H-SiC as an example, its advantages include a Mohs hardness second only to diamond, high thermal conductivity (~ 4.9 W cm^−1^ K^−1^), low intrinsic carrier concentration (~ 8 × 10^−9^ cm^−3^), and a wide bandgap (~ 3.26 eV) [9–11]. It has shown enormous application potential in fields such as new energy vehicles, rail transportation, and 5G communications(Fig. 1). The development of microelectronic devices and integrated micro-nano photonic devices has made the demand for high-yield and high-quality SiC wafer processing increasingly urgent. Traditional diamond or slurry-based wire sawing processes suffer from issues such as high material loss, low processing efficiency, and the introduction of defects like surface roughness and subsurface damage, often requiring expensive subsequent processing to compensate [12–16]. As SiC wafers continue to evolve toward larger sizes and thinner thicknesses, their mechanical strength decreases significantly, imposing stricter requirements on slicing technologies. Additionally, the inherent hardness and brittleness of SiC further exacerbate processing challenges. Consequently, the development of efficient, low-damage processing techniques has become a research hotspot in the industry. Ultrashort pulsed laser technology (with pulse duration shorter than 10^−12^ s) has garnered significant attention in the processing of hard and brittle materials such as SiC due to its high peak energy, narrow pulse duration, and benefits such as the absence of mechanical stress and non-contact processing [17–22]. Among these, laser vertical modification technology is considered the most advanced method for SiC wafer processing. Building on this, researchers continue to explore and innovate laser modification technology, aiming to improve processing efficiency while achieving micro-nano scale laser modification, thus overcoming technical bottlenecks such as poor process stability and severe material loss in laser modification and delamination.

This review systematically summarizes the research progress and key challenges of laser-SiC slicing technology. It comprehensively discusses the interaction mechanisms between laser and wide-bandgap semiconductor materials, key laser modification techniques, and process optimization strategies. The review aims to provide theoretical guidance and technical support for the further optimization and large-scale application of SiC wafer processing technologies.

## Interaction mechanisms between laser and wide-bandgap semiconductor materials

**Fig. 2:**
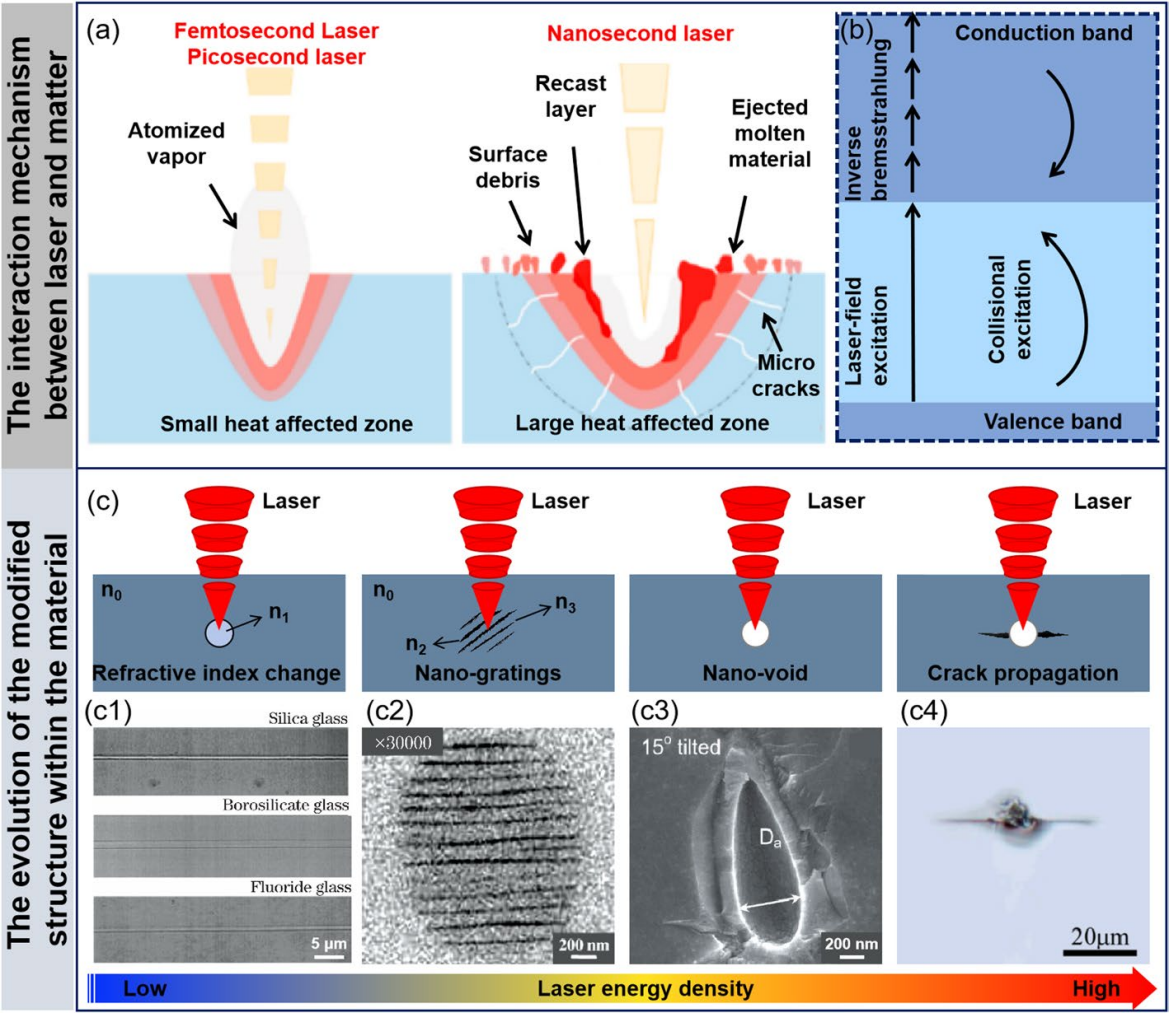
The interaction mechanism between laser and wide-bandgap semiconductor materials and the internal structural changes. **a** Laser-material interaction process [29] **b** The interaction process between laser and wide bandgap semiconductor materials [33–36] **c** The evolution of the modified structure within the material (**c1**) Waveguide structure directly written via ultrafast laser induced refractive index change [38] (**c2**) Back-scattered electron images of nanograting structures [39] (**c3**) Cross sectional electron microscopy image of nanovoid sturctures in sapphire [40] (**c4**) Crack propagation caused by laser internal modification structure [37]

The interaction between laser and wide-bandgap semiconductor materials is determined by both the laser characteristics and the intrinsic properties of the material. Nanosecond lasers (10^−9^ s), with pulse duration much longer than the material’s thermal relaxation time (10^−12^–10^−9^ s), generate a relatively large heat-affected zone (HAZ) during processing. This makes them suitable for efficient processing of large-sized wafers. However, this longer pulse duration prone to thermal-induced defects such as micro-cracks and re-cast layers. Compared to long-pulse lasers, ultrashort pulsed lasers such as femtosecond lasers (10^−15^ s) and picosecond lasers (10^−12^ s) feature shorter pulse duration and higher peak power, leading to fundamentally different interaction mechanisms with materials [24–26]. The interaction process between ultrashort pulsed lasers and materials is more complex, exhibiting clear nonlinear and nonequilibrium characteristics, with time and spatial scales spanning multiple orders of magnitude. In-depth research into the interaction process of ultrashort pulsed lasers with materials is crucial for understanding the mechanisms of material removal at subwavelength scales [27–29] (Fig. 2a).

In addition, based on the distribution characteristics of the electronic states in materials, they can be divided into two typical categories. One category is where most of the electrons are in a free state, such as in metallic materials; the other category is where most of the electrons are in a bound state, such as in dielectric and semiconductor materials. The difference in these electronic states leads to different mechanisms of laser-material interaction. When ultrashort pulse lasers interact with semiconductor materials, electrons in the bound state absorb photon energy and gain sufficient kinetic energy to transition from the valence band to the conduction band, becoming free electrons. The main absorption processes include linear ionization and nonlinear ionization. Nonlinear ionization encompasses multiphoton ionization, avalanche ionization, and tunneling ionization [18, 30] (Fig. 2b). Because semiconductor materials are transparent and brittle with almost no free electrons, the free electrons generated during the ultrafast laser nonlinear ionization process serve as seeds for avalanche ionization. Specifically, at low laser intensities, seed electrons, under the acceleration of the laser field, collide with atoms and ionize, generating secondary electrons, which increases the number of free electrons in the conduction band. Continuous collision ionization causes the free electron population to grow exponentially, triggering avalanche ionization. When the photon energy is less than or equal to the semiconductor bandgap, single-photon or multiphoton absorption ionization occurs. At high laser intensities, multiphoton absorption ionization becomes significant, where valence band electrons absorb multiple photons simultaneously and transition to the conduction band. As the laser intensity increases further, electrons are prone to tunneling effects, transitioning directly from the valence band to the conduction band, resulting in tunneling ionization [31–33] Thus, the electronic states of different materials play a decisive role in the laser-material interaction mechanisms. The bound electron characteristics of wide-bandgap semiconductor materials make their processing mechanisms more complex and exhibit significant nonlinear characteristics, providing a theoretical foundation for the subsequent laser processing research of wide-bandgap semiconductor materials.

In summary, the interaction mechanisms between lasers and wide-bandgap semiconductor materials are determined by both the laser characteristics (such as pulse duration, energy intensity, etc.) and the intrinsic properties of the material (such as electron state distribution and bandgap characteristics). When pulsed lasers are used for internal modification of transparent, hard, and brittle materials (such as SiC, sapphire, etc.), the interaction between laser energy and the material induces a series of structural changes, including refractive index variations, the formation of periodic nanograting structures, the generation of nanoholes, and crack propagation [34–40] (Fig. 2c). These structural changes are closely related to the laser parameters and material characteristics, providing a theoretical foundation for a deeper understanding of the laser-SiC modification process and laying the groundwork for further optimization of laser processing technologies.

## Laser-SiC slicing process flow and modification mechanism analysis

### Laser-SiC slicing process flow

**Fig. 3:**
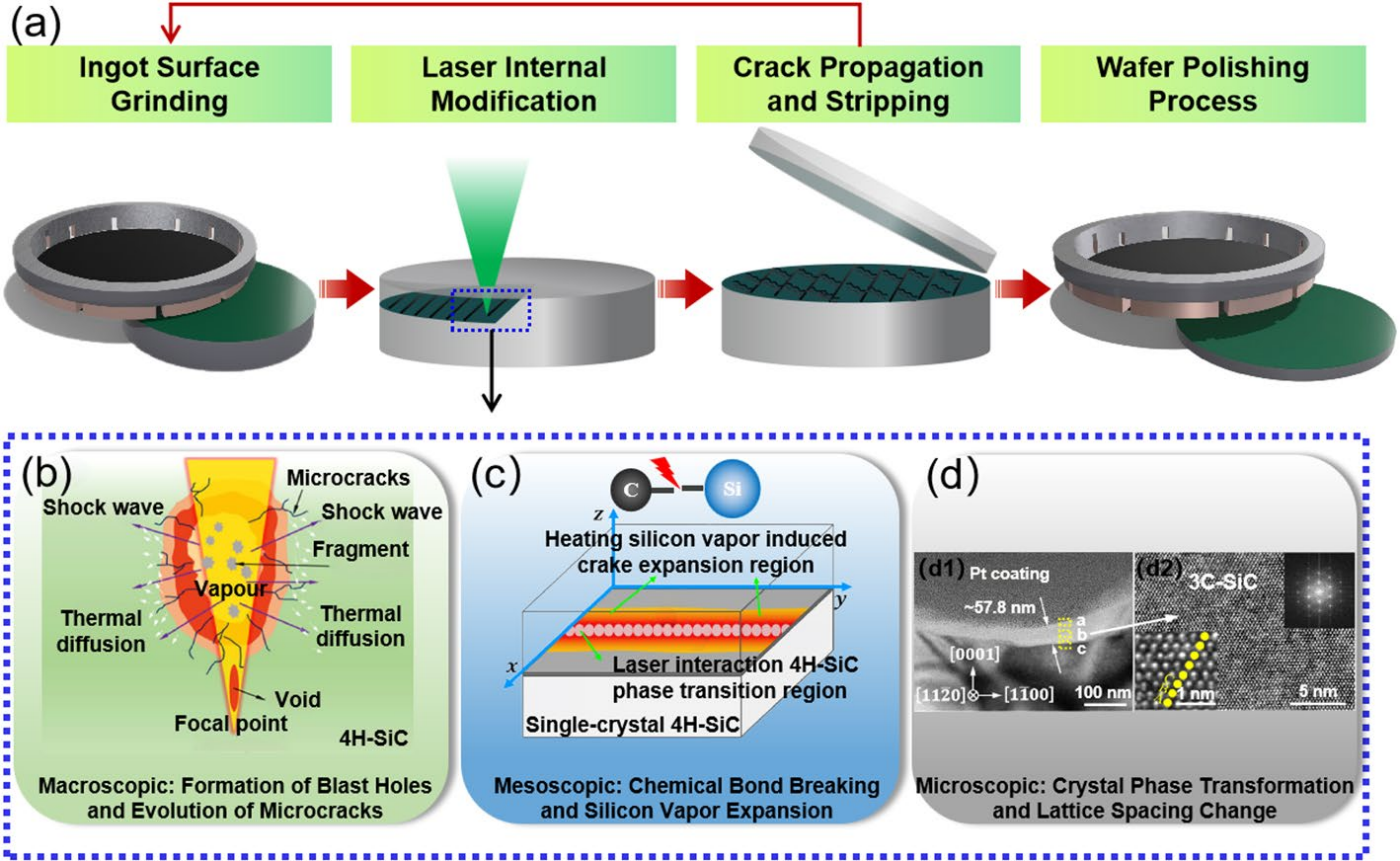
Wafer manufacturing process and modification mechanisms. **a** Schematic diagram of laser modification and stripping process flow **b** Modification mechanism: formation of macroscopic blast holes [42] **c** Modification mechanism: chemical bond breaking and silicon vapor expansion [44] **d** Modification mechanism: crystal phase transformation and lattice spacing change (**d1**) Bright-field TEM image of laser polishing [42] (**d2**) Corresponds to the detailed view at higher magnification of the b region in (**d1**) [42]

Laser-SiC slicing technology, as an innovative integration of laser precision processing and crystal stripping techniques, provides an effective solution for processing high-hardness, high-brittleness, and costly single-crystal materials like SiC. The laser modification and stripping process for SiC wafers is shown in Fig. 3a. In the laser modification stage, the laser beam is guided and focused inside the SiC wafer. The laser energy density outside the focal point is below the material’s damage threshold; therefore, a modification layer with a micro-nano scale thickness is selectively formed only at predefined positions inside the wafer. This modified layer contains microcracks and other micro-defects, effectively reducing the material’s bonding strength and improving the controllability of the detachment process.

In the cracking and stripping stage, after laser modification, external forces or chemical etching are applied to the wafer to induce stripping along the modified layer. The surface of the wafer stripping is then polished to remove the laser-modified layer and subsurface defects. This process is repeated, continuously producing high-quality wafers from the same crystal ingot. The wafer stripping surface must undergo successive processes such as mechanical grinding and chemical mechanical polishing (CMP) until the wafer surface is smooth and flat, meeting the requirements for subsequent use.

### Laser-SiC modification mechanism analysis

The laser modification process of SiC materials involves key scientific issues such as the nonlinear absorption of laser energy by the material, the evolution of internal microstructures, and the influence of laser light field control on material modification mechanisms. A deep exploration of these issues is crucial for optimizing the laser modification and stripping process, as well as improving the yield of laser slicing products.

- Macroscopic: Formation of explosive holes and evolution of microcracks

In the nanosecond laser modification process, thermal effects dominate. When the laser intensity exceeds the ablation threshold of SiC material, the modified region undergoes vaporization, forming “small hole”-like structures, i.e., capsule-shaped explosive pore, as shown in Fig. 3b. These consist of an explosion region and voids formed by the laser’s instant high-temperature focus, representing a key mode of heat transfer [41]. During this process, the four states of matter (solid, liquid, gas, and plasma) interact, and the accumulation of instantaneous heating leads to the formation of explosive holes and microcracks. Complex multiphysical evolution phenomena occur within the explosive holes, including energy-driven phase transitions and volume changes, temperature-driven crystal growth and material separation, as well as mass redistribution and material state fixation processes.

Specifically, the evaporation of material inside the small hole causes the hole to expand downward along the laser direction. The vapor pressure leads to the dynamic collapse and closure of the hole, which affects the reflection and refraction paths of the laser. The molten material flows due to recoil pressure and re-solidifies at the hole’s edge, forming a special disordered structure. Multiple reflections of the laser within the hole increase the interaction between the laser and the material. Additionally, due to the extremely high cooling rate (> 10^3^ °C/s), heat diffusion and convection occur above the focal point, and the rapid heating/cooling cycle generates a strong thermal gradient, forming metastable chemical, structural, and mechanical states. This promotes the propagation of microcracks in a chain-like or network microcracks. After multiple laser scans, the modified layer is ultimately formed. From a lateral view, the direction of microcrack propagation clearly depends on the crystal axis and aligns with the $\:\left[11\stackrel{-}{2}0\right]$ direction, with the modified layer being perpendicular to the surface normal. This orientation is beneficial for the crystal to delaminate along the modified layer after laser modification [42].

b. Mesoscopic: chemical bond breaking and silicon vapor expansion

In the laser modification of SiC materials, temperature-induced bond breaking is another key mechanism driving wafer modification and crack propagation, as shown in Fig. 3c. 4H-SiC has specific thermodynamic properties, with a sublimation temperature range of 2073–2273 K, a melting point of 3100 K, and a decomposition temperature as high as 3500 K. Under the influence of pulsed laser, the lattice temperature of 4H-SiC can exceed 3200 K [43, 44], surpassing its melting and sublimation temperatures, causing SiC to evaporate and melt, forming both liquid and gas phases of SiC. During the laser scanning modification process, the thermal accumulation effect is significant, and the internal temperature can reach up to 3500 K. As the liquid phase gradually transitions into a metastable Si-C system, SiC decomposes into amorphous Si and amorphous C.

Through the complex physical processes described above, the modification and stripping of SiC wafers are achieved, involving three key stages: the generation, growth, and interconnection of cracks [86]. When the pulsed laser is focused on the material, energy is rapidly deposited, causing a rapid temperature increase at the focal point. The subsequent rapid heating/cooling cycle creates a significant temperature gradient, leading to the distribution of compressive and tensile stresses at different locations within the material. When these stresses exceed the material’s ultimate strength, microcracks are generated. It is important to note that the threshold for microcrack formation and the direction of propagation are closely related to the atomic arrangement of SiC material. On the other hand, under high-temperature conditions, SiC melts and evaporates, with amorphous Si transforming into expanding Si vapor, triggering micro-explosions. These micro-explosions generate pressures up to several hundred GPa within SiC [45], compressing the microcrack walls and promoting further crack propagation [46]. As the microcracks continue to expand and evolve, they eventually interconnect, facilitating the separation of the wafer.

c. Microscopic: crystal orientation transition and lattice spacing changes

From the perspective of crystal structure transformation, a series of physicochemical changes triggered by pulsed laser irradiation also include crystal orientation transitions and lattice spacing changes, as shown in Fig. 3d. The stable Si-C bonds in the 4H-SiC phase (α-SiC) dissociate at high temperatures, leading to elemental orientation separation. The disordered carbon layers gradually transform into a layered structure with expanded crystallinity, driving the reconstruction of the SiC material and ultimately leading to the formation of the 3 C-SiC phase (β-SiC) [47]. Through Inverse Fast Fourier Transform (IFFT) analysis, it was found that the lattice spacing of the amorphous region (d = 0.261 nm) is significantly larger than the initial lattice spacing of 4H-SiC (d ≈ 0.251 nm). Furthermore, after the formation of the 3 C-SiC phase, its lattice spacing (d = 0.308 nm) is also noticeably larger than that of 4H-SiC. This increase in lattice spacing is primarily attributed to the residual thermal stress generated during the formation of the modified layer during laser processing, and is also one of the key factors driving microcrack formation [42].

Based on the above analysis, the pulse laser modification of SiC materials is a complex process involving multiple scales and physical fields. A deeper understanding of the modification mechanisms is crucial for the precise control of the modified layer, which is the foundation for further achieving high-quality micro-nanoscale modification. Future research should further integrate experimental and simulation approaches to quantitatively analyze the interactions between different mechanisms, providing more comprehensive theoretical guidance for the optimization and innovation of laser modification processes.

## Optimization of laser-SiC cutting process

The quality of internal modification, the thickness of the modified layer, and its uniformity directly affect the difficulty and efficiency of subsequent peeling and polishing processes. Therefore, further optimization of the processing technology is crucial. By adopting a multidimensional process optimization approach, such as material pre-treatment, modification process optimization, and improvements in the peeling process, wafer yield can be enhanced while reducing processing costs and time, thereby meeting the requirements for industrial application.

### Material pre-treatment process optimization

The crystal defects of SiC ingots (such as dislocations, twins, impurities, etc.) and the non-uniformity of doping concentration can lead to variations in resistivity distribution. These factors directly affect the absorption of laser energy and the formation of the modified layer, significantly influencing the effectiveness of the laser modification process.

In addition, macroscopic features such as surface roughness can interfere with the focusing performance of the laser inside the material and the modification effect, potentially causing uneven energy reflection and absorption, which in turn affects the uniformity and stability of the modified layer. Prior to modification, a comprehensive quality assessment and pre-treatment of the SiC ingot should be conducted, including defining the doping level and transmittance of the sample, calibrating the crystal orientation, and measuring the surface roughness.Therefore, a comprehensive quality assessment and pre-treatment of SiC ingots should be performed before modification, including testing the sample doping level and transmittance, calibrating the lattice direction, and changing the surface roughness.

#### Influence of sample transmittance

**Fig. 4:**
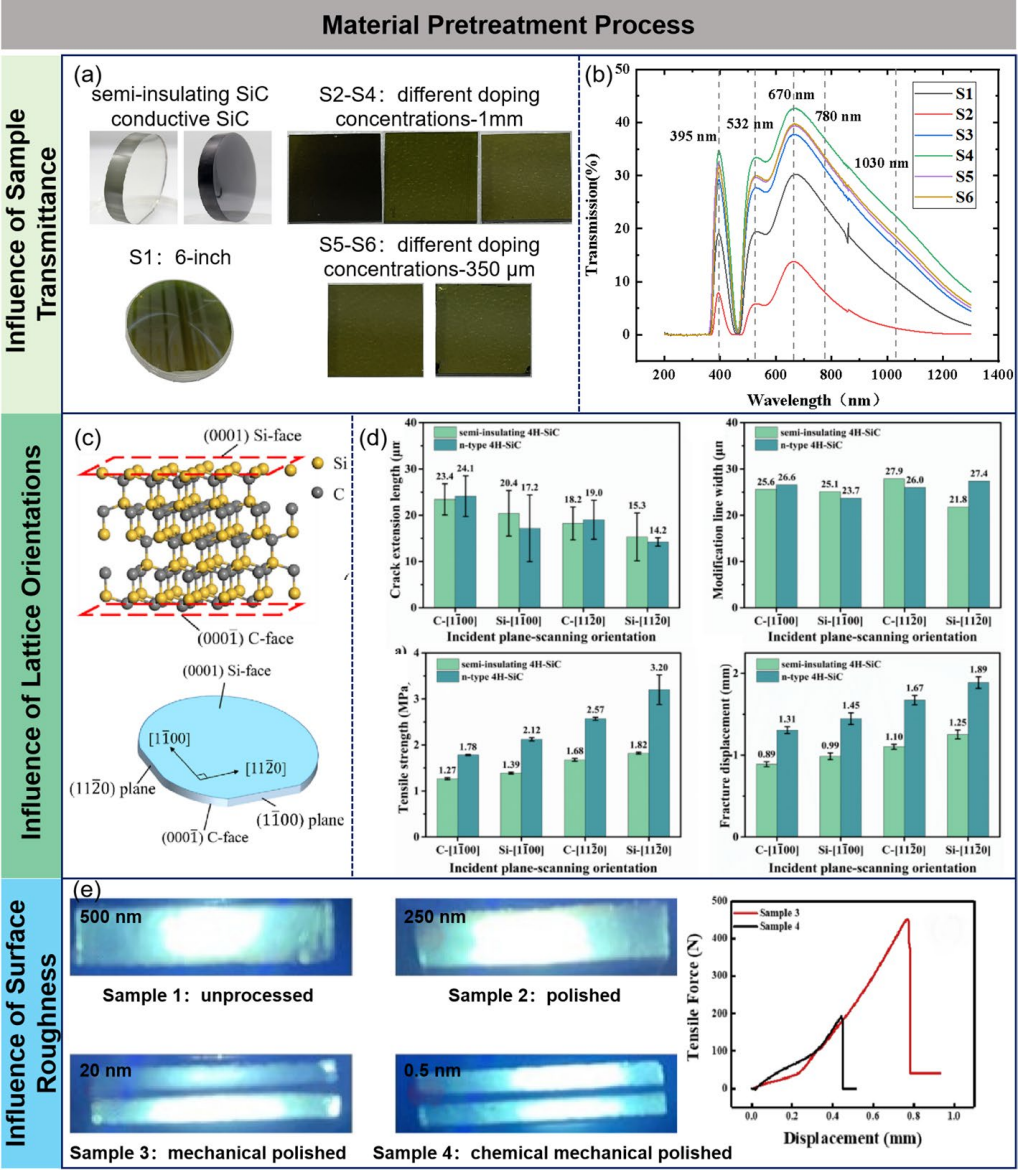
Laser-SiC slicing process flow and modification mechanism. **a** SiC samples with different doping concentrations and thicknesses **b** Transmittance of SiC samples **c** Crystal structure and crystal orientation of 4H–SiC **d** Crack extension lengths, modification line durations, tensile strength and fracture displacement of different processing scenarios **e** Cross-sectional views of the modified layers of samples with different roughnesses and the tensile force relationships of the samples [66, 67, 74]

Based on their electrical properties, SiC materials can be classified into two categories: semi-insulating (with resistivity > 10^5^ Ω cm) and conductive (with resistivity < 0.1 Ω cm) [48–50]. Their optical characteristics exhibit a significant correlation with doping concentration, (Fig. 4a). Semi-insulating SiC typically demonstrates high transmittance (with visible light transmittance > 70%), and the crystal surface appears transparent or light yellow. In contrast, conductive SiC, due to the band modulation effects of doping elements such as nitrogen (N) or aluminum (Al), experiences a red shift in the material’s absorption edge, leading to a significant reduction in transmittance (visible light transmittance approximately 30%) and a dark green or dark gray appearance. Notably, even within the same type of SiC ingot, variations in doping concentration caused by fluctuations in crystal growth processes can result in color gradients, leading to batch-to-batch transmittance fluctuations (differences up to ± 15%) [51–58]. This optical non-uniformity directly affects the energy absorption efficiency and thermal distribution during laser processing. Therefore, real-time monitoring of the sample and dynamic adjustment of laser parameters (such as energy density, pulse width, and scanning strategy) are necessary to optimize the quality of the modified layer.

In addition, an effective modification process involves focusing the laser on a specific internal region of the material while avoiding surface absorption and subsequent damage. Selecting a laser with an appropriate transmittance as the light source facilitates internal laser focusing and ensures stable modification. Through transmittance testing of different samples (Fig. 4b), it is evident that 4H-SiC exhibits high transmittance at the wavelengths of 395 nm, 532 nm, and 670 nm. Although the transmittance at 395 nm and 650 nm is even higher, the output stability of existing lasers at these wavelengths is relatively inferior. Therefore, considering both processing stability and modification quality, lasers operating at 532 nm and 1030 nm have emerged as the predominant choices for the modification processing of 4H-SiC.

#### Influence of lattice orientations

Single-crystal SiC wafers are anisotropic, with two inequivalent faces: the Si face ((0001) face) and the C face ((0001̅) face), as shown in Fig. 4c. The primary plane of 4H-SiC wafers is the (11̅00) plane, and the secondary plane is the (112̅0) plane, with crystal orientations of [11̅00] and [112̅0], respectively [59−61]. In the field of SiC machining, studies have shown that crystal orientation and crystal plane have a great influence on the machining process[62−65]. Therefore, understanding the influence of crystal orientation on the laser internal modification process will help further optimize the modification process [66]. Yao et al. found that during laser modification, crack propagation length depends on crystal orientation and incident plane, following the order: C-[11̅00] > Si-[11̅00] > C-[112̅0] > Si-[112̅0] (Fig. 4d). The modification line duration, however, shows no significant relation to the crystal orientation or incident crystal plane. Additionally, the peeling tensile strength is significantly affected by the crystal orientation and incident crystal plane, with the [11̅00] orientation showing lower peeling tensile strength. Surface morphology and roughness after laser cutting vary by crystal orientation, with [11̅00] yielding better results. Ultimately, C-[11̅00] was identified as the optimal orientation for successful peeling modification of a 6-inch n-type 4H-SiC wafe [67].

#### Influence of surface roughness

The core of laser modification and delamination technology lies in forming a modification layer inside the SiC ingot using a laser, thereby reducing crystal bonding strength and enabling efficient delamination. However, during the laser modification process, the surface roughness of the ingot directly impacts laser scattering and the uniformity of the modified layer. Excessive surface roughness can lead to increased laser scattering, reducing modification efficiency and potentially causing surface damage to the ingot [68, 69].

Therefore, prior to laser modification, the ingot surface must undergo polishing pre-treatment to control roughness at the nanometer level (e.g., Ra ≤ 5 nm), optimizing laser interaction and enhancing modification precision and stability. Common surface pre-treatment methods, such as mechanical polishing, laser polishing, and chemical polishing, effectively improve surface quality and ensure high-precision processing requirements [70–73].

Wang et al. found that surface roughness of SiC significantly affects laser processing results [74]. Samples with different roughness levels (500 nm, 250 nm, 20 nm, and 0.5 nm) exhibited varying outcomes after laser treatment, as shown in Fig. 4e. Samples with rough surfaces (500 nm and 250 nm) experienced noticeable ablation during the laser process, mainly due to unstable reflection and absorption of laser energy caused by the rough surface. In contrast, smooth surfaces (20 nm and 0.5 nm), treated by mechanical or chemical-mechanical polishing, allowed for more uniform laser energy absorption, leading to the formation of stable and uniform modified layers, significantly reducing surface damage. Further experiments by Wang et al. showed that samples with lower surface roughness exhibited lower separation tensile force in stretching tests, confirming that better surface quality reduces energy loss, improves laser modification effectiveness, enhances the uniformity of the modified layer, and promotes stable micro-crack propagation.

In summary, the pre-processing steps in laser modification are crucial for ensuring the stable formation of the modified layer. By selecting an appropriate laser wavelength based on the sample’s transmittance, precisely determining the processing direction according to the crystal lattice orientation, and applying surface pretreatment to the sample, the absorption efficiency of laser energy can be significantly enhanced. This promotes the uniform formation of the modified layer and reduces the likelihood of unnecessary cracks and other potential processing defects during laser modification. These steps provide a foundational guarantee for subsequent processing and further advance the optimization of the modification and stripping processes.

### Modification and peeling process optimization

#### Modification quality optimization

The core of modification quality optimization lies in precisely controlling the modified layer. However, the nonlinear effects induced by ultrafast lasers often lead to excessive extension of the modified layer and the formation of strong bonding forces between modification zones, presenting significant challenges for subsequent peeling processes. To address this issue, existing studies have proposed three main optimization strategies: (1) optimizing the laser pulse duration to control the modification process [37, 42, 75–77], (2) using dual-pulse or multi-pulse trains for precise modification [78–84], and (3) implementing modification strategies based on multi-parameter regulation [37, 85].

- Strategy 1: modification control based on pulse duration

**Fig. 5:**
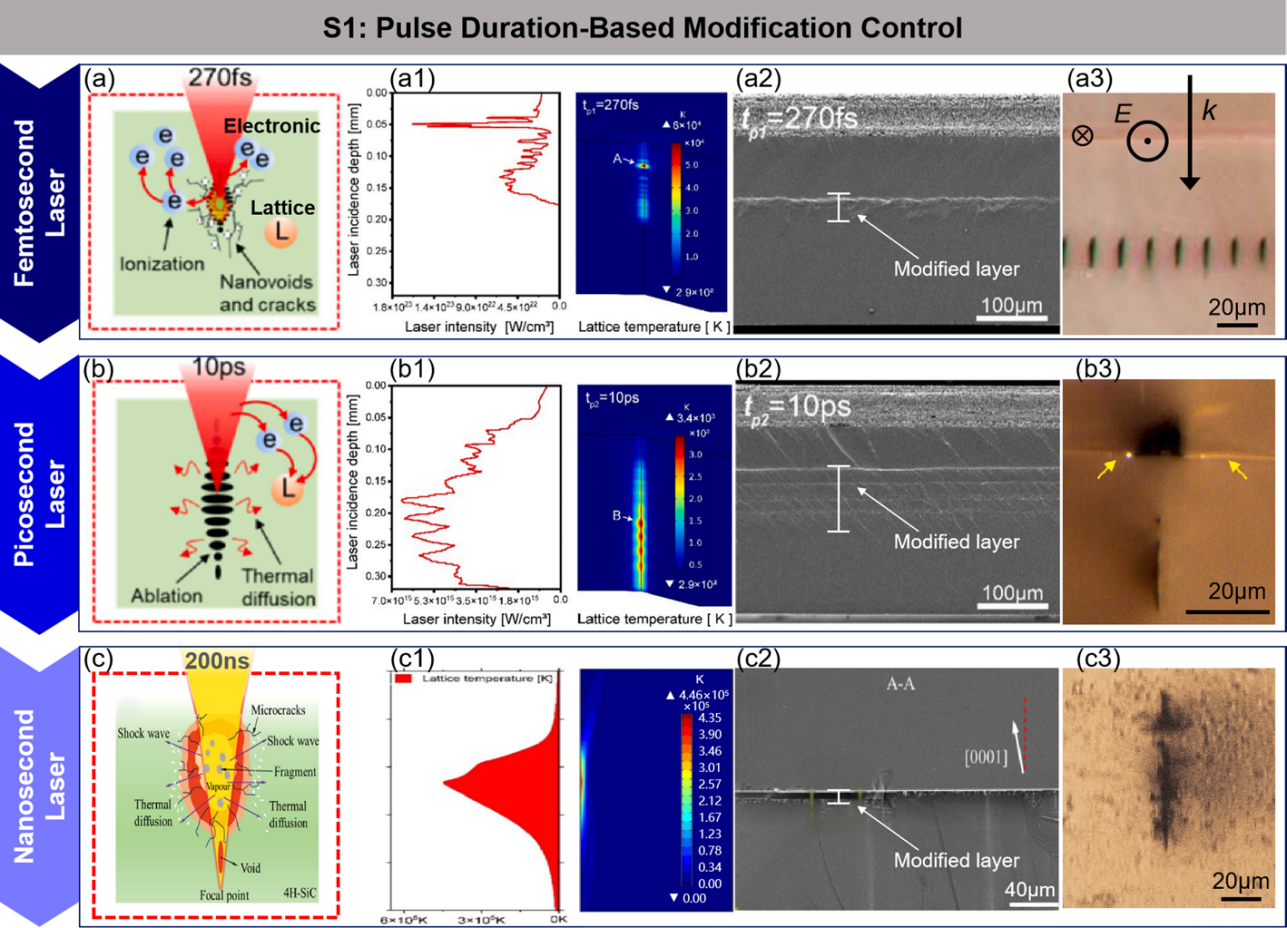
Modification regulation based on pulse duration. **a**–**c** Schematic diagram of femtosecond, picosecond and nanosecond laser modification [42] (**a1**–**c1**) Femtosecond, picosecond and nanosecond pulsed laser simulation results: energy deposition and transient lattice temperature field distribution along the laser incident axis [75, 76] (**a2**–**c2**) Femtosecond, picosecond and nanosecond laser modified layer structure [42, 75] (**a3**–**c3**) Femtosecond, picosecond and nanosecond laser modified spot structure [77]

Pulse duration is a key parameter in laser modification. Its selection not only determines the energy deposition method and efficiency in the material but also directly affects the intensity of nonlinear effects as well as the structure and quality of the modified layer, as shown in Fig. 5.

Femtosecond lasers, with their highly concentrated pulse energy in both time and space, can complete energy deposition before the electron-lattice interaction occurs, significantly reducing the impact of heat diffusion on processing accuracy. However, at lower energy densities, femtosecond lasers tend to form unstable and discontinuous modified layers. As the energy density increases, nonlinear effects can cause multi-focusing issues, leading to an elongated modification region. In contrast, picosecond lasers, with relatively longer pulse duration, provide more time for interaction within the material, allowing for more effective induction of structural changes and enabling processing of larger areas within the same time frame, significantly improving processing efficiency. However, their ultrashort pulse characteristics still induce certain nonlinear effects, which need to be controlled during the processing [75–77]. For nanosecond lasers, the pulse duration is much larger than the electron-phonon coupling time of SiC, with the modification mechanism primarily driven by thermal effects. The plasma generated within the material causes thermal shock waves, forming high-density dislocation layers. Subsequent pulses overlap with these dislocation layers, further promoting the expansion of microcracks. This mechanism can efficiently achieve modification in SiC wafer slicing [42].

**Fig. 6:**
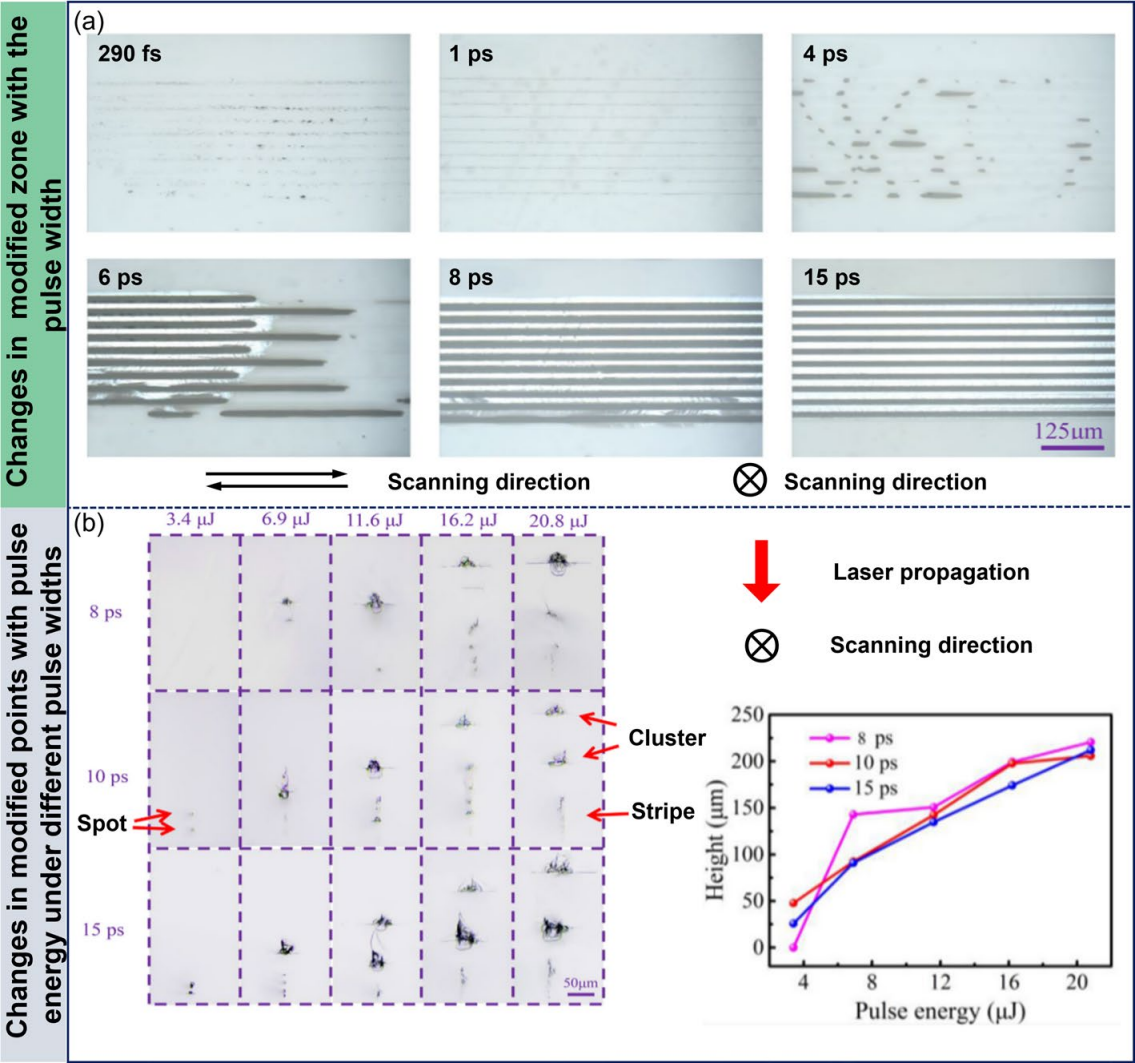
Structural changes under different pulse durations and pulse energies. **a** Top view of the modified layer inside the silicon carbide under different pulse duration **b** The cross-sectional morphologies of modified structure at different energies at different pulse duration; The relationship between the height of modified layer and pulse energy at different pulse duration [37]

To systematically study the impact of pulse duration on modification effects, Zhang et al. used an ultrashort pulsed laser with a central wavelength of 1030 nm and adjustable pulse durations (290 fs to 15 ps) [37]. Their study revealed the effects of pulse duration on SiC modification structures, as shown in Fig. 6a. Under a 290 fs ultra-short pulse, only slight ablation occurred on the material surface, with no significant modification inside. This phenomenon was mainly attributed to strong nonlinear effects such as multi-photon absorption, Kerr-induced phase distortion, strong plasma shielding, and defocusing, which prevented SiC from absorbing enough energy for modification. As the pulse duration increased to 1 ps, no ablation occurred on the surface, and a modification line with a duration close to the spot diameter formed inside, indicating a reduction in nonlinear effects and a significant improvement in energy deposition efficiency. When the pulse duration was increased to 4 ps, black spots and short modification lines with a duration of about 30 μm appeared, indicating that the modification structure was gradually stabilizing. Further increasing the pulse duration to 6 ps, the short modification lines became longer and were accompanied by white areas, which were caused by crack formation leading to light reflection, showing that the increase in pulse duration effectively promoted crack propagation. Finally, when the pulse duration reached 8 ps and above, continuous modified regions and cracks were stably formed across the entire processing area, indicating that longer pulse durations significantly weakened nonlinear effects, stabilizing the modification structure and crack formation, thus achieving high-quality modification.

Additionally, the team studied how the modification structure varied with pulse energy at different pulse durations (8 ps, 10 ps, 15 ps), as shown in Fig. 6b. The results revealed that for a fixed pulse duration, the modification layer height increased significantly with higher pulse energy, as higher energy triggered physical and chemical reactions such as atomic rearrangement and phase transitions. At the same pulse energy, longer pulse durations required less energy to achieve modification, indicating that longer pulses more efficiently induce modifications. This phenomenon is closely related to the reduction of nonlinear effects at longer pulses, leading to more stable energy deposition and a more controllable modification process.

In contrast, the modification mechanism of nanosecond long-pulse lasers is based on thermal effects, which allow for higher material removal rates during slicing. Additionally, nanosecond laser systems are relatively simple, offering better stability and lower maintenance requirements. These characteristics help increase processing throughput, making them more suitable for large-scale wafer processing where cost control and efficiency improvement are essential. For example, Li et al. used a laser with a center wavelength of 532 nm and a pulse duration of 200 ns to modify and peel single-crystal SiC [42]. The study showed that the modification layer formed inside the SiC had an average thickness of 6.15 μm (with a total thickness of 13.67 μm including the peeling region), with microcracks running through the layer, and successful wafer peeling was ultimately achieved.

b. Strategy 2: modification control based on multi-pulse trains

In the ultrashort-pulse laser modification process of SiC, nonlinear effects can cause uneven energy absorption and deposition, leading to fragmentation of the modified layer, with difficulty in precisely controlling its lateral and longitudinal dimensions. Moreover, excessive elongation of the laser propagation direction significantly affects the quality of wafer peeling.

**Fig. 7:**
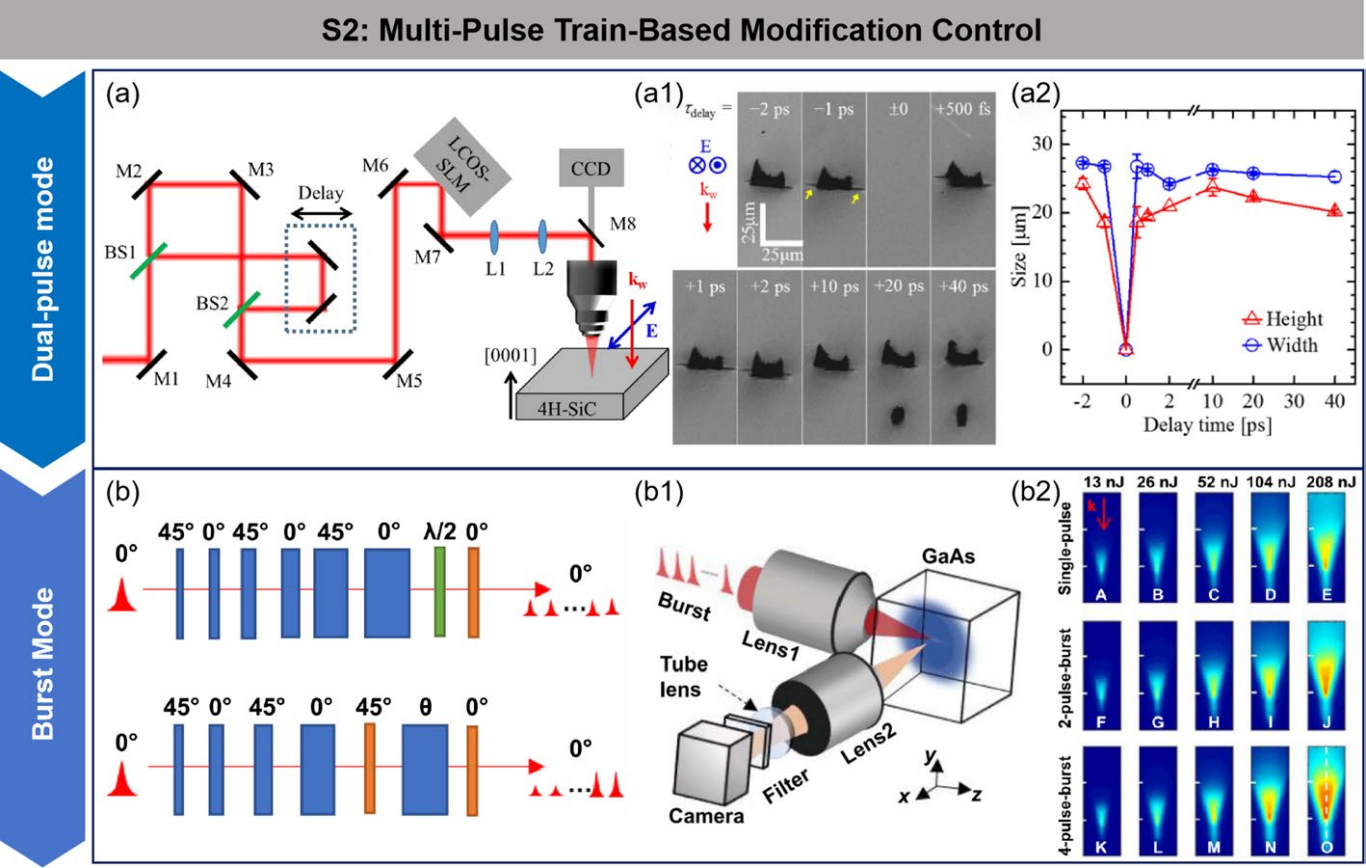
Modification regulation based on double pulses mode and burst mode. **a** Schematic of experiment setup with double-pulses configuration (**a1**) Cross-sectional view of the modified region formed in a 4H-SiC under femtosecond double-pulses irradiation, with yellow arrows indicating crack locations (**a2**) Relationship between the size of the internal modified structure in 4H-SiC and the double-pulses delay time [78] **b** Schematic diagram of the burst mode pulse train based on polycrystals (**b1**) Experimental setup diagram (**b2**) The luminescence map excited by different pulse energies (written on the top) and different trains of pulses (written at the left) [84]

To address the above issues, researchers have proposed the use of dual-pulse/pulse-train techniques to suppress nonlinear effects. By precisely controlling the time delay and energy distribution between pulses, the dynamics of laser-material interaction are optimized. Firstly, the plasma effect induced by the initial pulse can modulate the material’s local optical properties, optimizing the propagation path and energy absorption distribution of subsequent pulses, thus preventing excessive elongation of the modified layer along the laser propagation direction. Secondly, the dual-pulse configuration extends the electron-photon interaction time, enabling stable energy deposition within the material, significantly improving the homogeneity and structural stability of the modified layer. Kim et al. investigated the modification results of SiC wafers using femtosecond laser dual-pulses [78–81]. As shown in Fig. 7a–a2, the experiment employed a femtosecond laser with a central wavelength of 780 nm, pulse duration of 220 fs, and repetition frequency of 1 kHz, combined with a Mach-Zehnder dual-pulse optical setup. By precisely adjusting the time delay between pulses and the total pulse energy, and using a spatial light modulator (SLM) to compensate for spherical aberrations, they found that the dual-pulse configuration significantly suppressed the nonlinear effects induced by ultrashort pulses. This optimization improved the structure of the modified layer, reducing its thickness and promoting the directional expansion of cracks on both sides of the modified layer. Based on this optimization, the peeling of the SiC wafer was successfully achieved with a peeling surface roughness of less than 5 μm. The lower roughness further confirmed the reduction in the modified layer thickness, while the separation force in the dual-pulse experiment was significantly lower than in the single-pulse experiment. This study provides an innovative technical solution for high-precision, low-damage processing of SiC wafers.

Subsequently, Hirata et al. from DISCO Corporation proposed the Amorphous Black Repeat Absorption (KABRA) process based on the dual-pulse principle [82]. This technology uses ultrashort pulse lasers to form a thermal decomposition layer inside the crystal ingot, and by increasing the number of absorption layers, the plasma morphology becomes flatter, creating a unique KABRA layer. This leads to more uniform stress along the cleavage plane direction, promoting precise expansion of horizontal cracks. The results showed that the total thickness variation of the wafers produced using the KABRA process was 5.0 μm, with a warp of 1.6 μm, and the material loss during slicing was only half of that of traditional methods. Additionally, samples peeled using the high-quality modified layer did not require the grinding step typically needed in conventional processes, further improving the quality of the wafer.

The aforementioned studies all use traditional geometric optics methods, such as the beam-splitting and recombination technique, to achieve dual-pulse output [83]. However, these conventional optical splitting methods have notable limitations: first, the difficulty in optical system collimation, which hampers processing stability; second, the large number of required optical components, resulting in a low system integration and occupying significant space, making industrial application challenging.

To address these issues, Wang et al. proposed an ultrafast laser writing technique based on terahertz (THz) repetition frequency pulse trains, as shown in Fig. 7b–b2 [84]. By using the polarization beam-splitting principle and precisely stacking YVO₄ birefringent crystals of different thicknesses, they converted the center wavelength of 1030 nm femtosecond laser (amplified to 1550 nm through an optical parametric amplifier) into a THz repetition frequency pulse train, enabling precise multi-pulse control. The significant advantage of this method is that, through the cooperative effect of the multi-pulse sequence, it effectively reduces the plasma density in the pre-focus region, allowing more energy to reach the focal area and thus improving processing efficiency. Experimental results show that the optimized 64-pulse square-wave pulse train can efficiently achieve internal modification of GaAs material, opening a new path for the fabrication of internal micro-nano structures in transparent materials. This successful application also provides a new approach for further optimization of the modified layer.

c. Strategy 3: modification control based on parametric synergy.

Building upon the optimization of pulse duration and pulse mode, further enhancement of the comprehensive performance in SiC wafer processing requires synergistic optimization of the process parameters during the modification process. By precisely adjusting key process parameters such as laser pulse energy, scanning speed, scanning interval, and scanning depth, the morphology of the modified layer can be further optimized, providing favorable conditions for the subsequent peeling process.

**Fig. 8:**
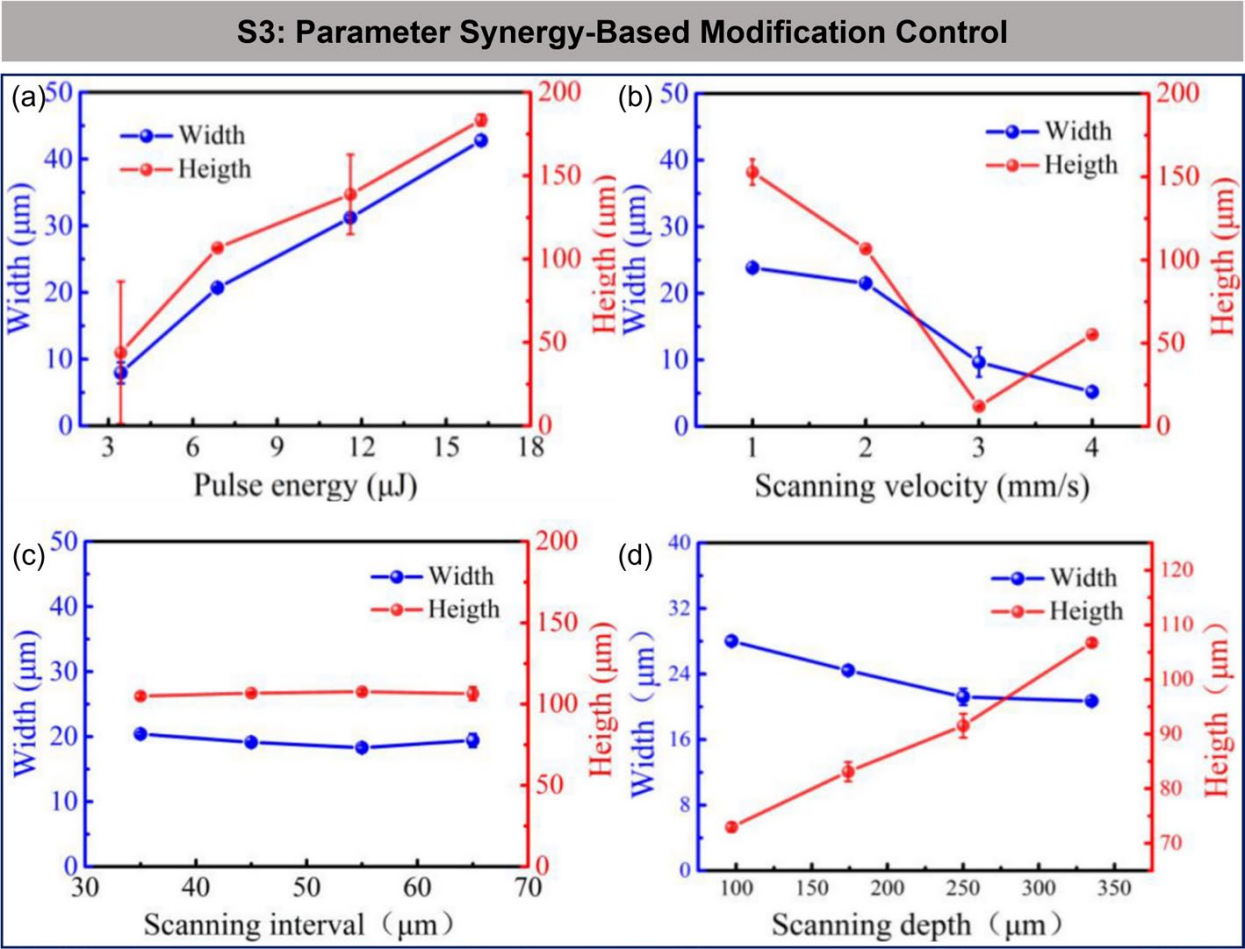
Modification regulation based on parameter collaboration [37]. **a** Effect of pulse energy on the morphology of modified layer **b** Effect of scanning velocity on the morphology of modified layer **c** Effect of scanning interval on the morphology of modified layer **d** Effect of scanning depth on the morphology of modified layer

Zhang et al. studied the relationship between the modification structure and processing parameters in ultrafast laser processing of SiC wafer slicing (Fig. 8) [37]. The results revealed the following trends regarding the influence of various process parameters on the modification effect: Increasing the pulse energy significantly enhances the number, thickness, and duration of the modified layer, but it also complicates the crack propagation path and increases the risk of surface ablation. Increasing the scanning speed reduces the thickness and duration of the modified layer, weakening the modification effect. The scanning interval mainly affects crack propagation; smaller intervals improve the quality of the stripping surface but increase tensile stress, while larger intervals facilitate stripping but may increase surface roughness. Increasing the scanning depth raises the height of the modified layer and reduces its duration, but does not change its basic structural type.

**Fig. 9:**
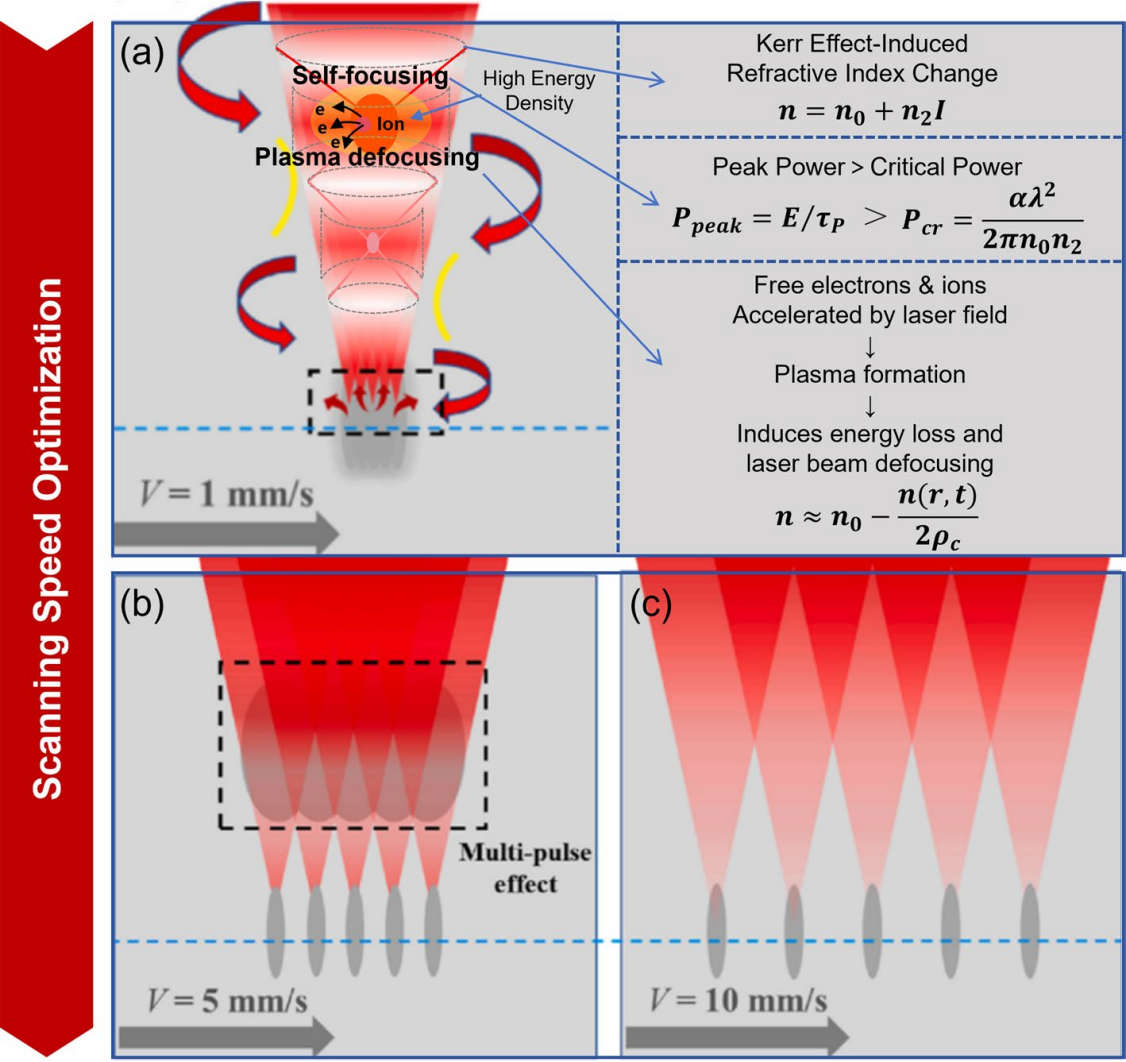
Regulation of the modified layer by scanning speed [85]. **a** Modified layer formation at v = 1 mm/s **b** Modified layer formation at v = 5 mm/s **c** Modified layer formation at v = 10 mm/s

In addition, controlling the scanning speed helps to further mitigate the elongation of the modified layer caused by nonlinear effects (Fig. 9) [85]. Changes in scanning speed influence the interaction time between the laser and the material, thereby modifying the extent of nonlinear effects. At lower scanning speeds (< 1 mm/s), the laser has a longer interaction time with the SiC material. As the high-power laser beam propagates, the Gaussian intensity distribution of the beam causes the center intensity to be higher than the edges. This induces a photo-induced refractive index change in the material due to the Kerr effect. The area with a refractive index change causes the laser to behave like it is passing through a convex lens, gradually focusing the beam. When the peak power at the focal point exceeds the critical power for self-focusing, plasma generated at the focal point causes beam defocusing. However, because the interaction time is long, the Kerr self-focusing effect dominates, and the laser beam continuously focuses and defocuses, creating a filament-like phenomenon. This leads to a multi-layer structure in the modified layer, with the layer elongating in the opposite direction of laser propagation. The number and height of layers increase as laser pulse energy increases. As the scanning speed increases (> 10 mm/s), the laser’s interaction time with the material is significantly reduced. The laser pulse energy is unable to deposit continuously, and although the peak power exceeds the critical power for self-focusing, the interaction time is too short for the self-focusing effect to fully develop, thereby suppressing the elongation of the modified layer.

In summary, the improvement of modification quality is a process of multidimensional collaborative optimization. Laser pulse duration, as a key parameter, directly affects the interaction mechanism between the material and the laser, making it the primary determinant of the modified layer’s depth, quality, and crack propagation controllability. Femtosecond, picosecond, and nanosecond lasers each have distinct characteristics, and their selection must balance cost and efficiency based on specific processing requirements. On this basis, precise control of process parameters such as laser pulse energy, scanning speed, scanning interval, and scanning depth can be precisely controlled, it is possible to further enhance the modification precision and compress the modified layer to the nanoscale.

#### Optimization of modification efficiency

**Fig. 10:**
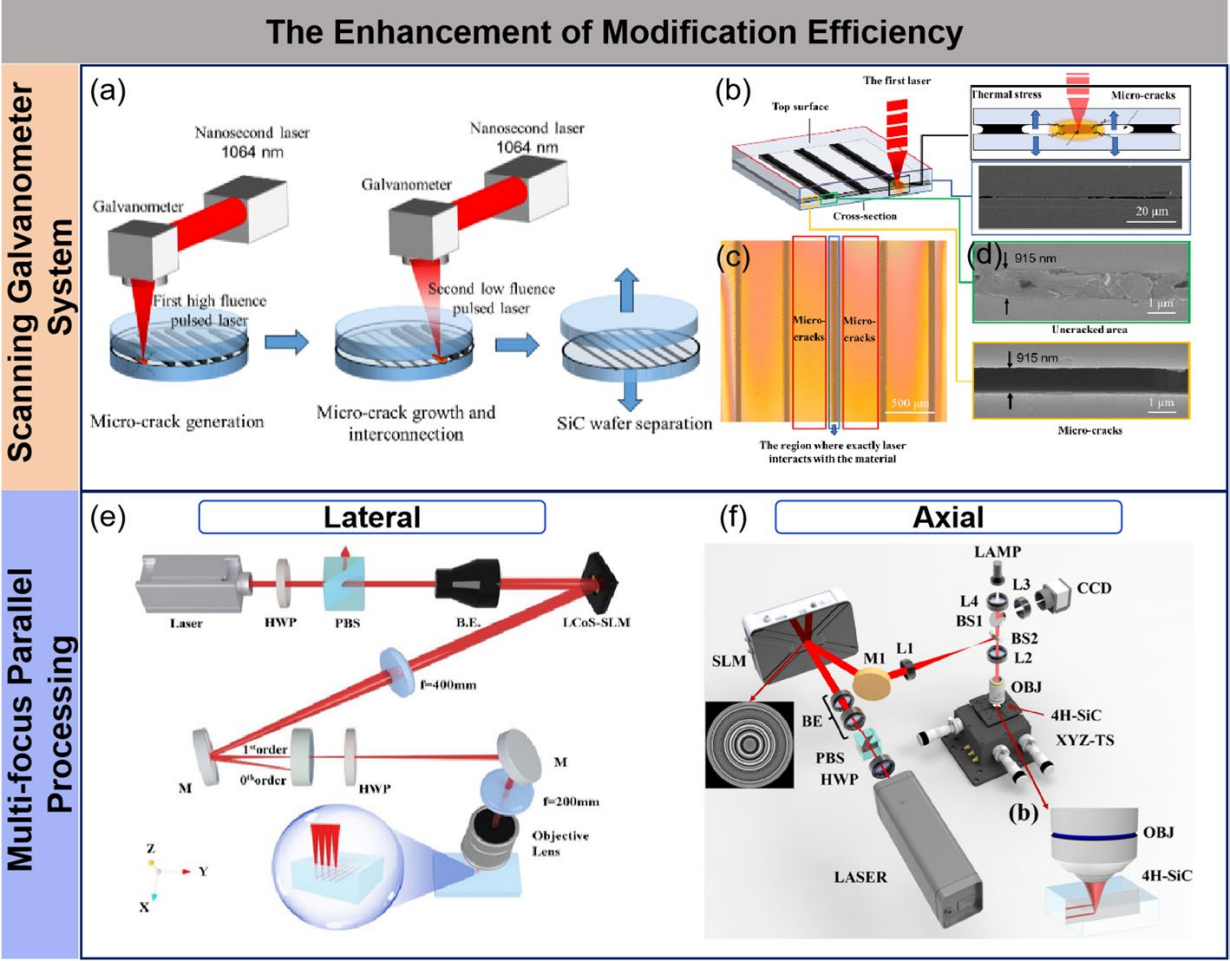
Improvement of modification efficiency. **a** Schematic of all-laser processing induced micro-cracks generation and growth manipulation **b** Mechanism of micro-cracks formed by the first pulsed laser ablation and cross-section view of micro-cracks inside SiC **c** Top view of SiC wafer with micro-cracks generation **d** Cross-section view of multiple micro-cracks and uncracked area inside SiC [86] **e** Schematic diagram of a lateral parallel processing device [90] **f** Schematic diagram of an axial parallel processing device [91]

To meet the technical demands of cost reduction and efficiency improvement, enhancing the modification efficiency of SiC wafers has become a key focus of current research. Traditional laser modification processes generally rely on an objective lens system combined with an electric displacement platform, where modification is carried out by scanning the focus point one by one. However, limited by the working distance of the objective lens, prolonged scanning can lead to issues such as surface contamination and focal shift due to thermal accumulation, which restrict the processing capacity of the lens. Therefore, the introduction of scanning galvanometer systems and telecentric lenses has become an effective solution to this problem. Telecentric lenses can correct the angular variations of the beam within the processing area, ensuring that the focal point remains perpendicular to the workpiece surface during large-size wafer processing, significantly improving both processing precision and stability. Liu et al. proposed a fully laser-based processing method for inducing and growing microcracks using dual-laser pulses (Fig. 10a–d) [86]. This method utilizes the first high-energy density pulsed laser (18–75 mm/s) to generate microcracks inside the SiC, enhancing laser energy absorption and weakening the material’s binding force. Subsequently, the second low-energy density pulsed laser rapidly scans (2000–3000 mm/s) to promote the growth and interconnection of these microcracks. After multiple scanning passes, the isolated microcracks gradually transform into a continuous network, ultimately forming a microcrack modification layer with a thickness of only 915 nm. This process ensures slicing quality and processing efficiency while significantly reducing kerf loss, enabling high-precision separation of SiC wafers. Moreover, the formation of a nanoscale modification layer marks a significant breakthrough in laser modification technology for SiC, paving the way for high-efficiency and low-damage processing.

Multi-focus parallel processing technology is another important method for improving laser modification efficiency. Its core principle is generating multiple focal points from a single laser pulse to achieve efficient modification of transparent and brittle materials. This technology not only improves laser energy utilization but also effectively shortens processing time. Common methods for generating multi-focus lasers include lens arrays, diffraction optical elements (DOE), and spatial light modulators (SLM) [76, 87–89]. In multi-focus parallel processing, the processing method can be divided into two directions: lateral and axial. Du et al. employed a lateral multi-focus picosecond laser vertical modification method for parallel processing of 6-inch SiC wafers, achieving efficient slicing (Fig. 10e–f). They analyzed the electric field distribution at focal points within the material using vector diffraction modeling under high numerical aperture conditions and developed a non-iterative computer-generated holography (CGH) method to address the issue of spherical aberration caused by the refractive index mismatch between the material and air [90]. This method allows for the generation of multi-focus points within the material with spherical aberration correction, enabling precise control over the position and number of the focal points. Experimental results showed that multi-focus technology not only reduced the energy at each focal point, mitigating the elongation of the modified layer caused by nonlinear effects, but also significantly promoted crack propagation. For instance, with four focal points, the modification efficiency increased fourfold compared to single-focus scanning, while the separation tensile strength dropped from 22.4 to 7.6 MPa. Additionally, the surface roughness after delamination was significantly reduced to the nanoscale, optimized from 1.3 μm to 432 nm. This process not only enhanced both modification efficiency and quality but also provided favorable conditions for the subsequent wafer polishing process.

Lu et al. employed an axial dual-focus laser processing method by incorporating spherical aberration correction into the traditional weighted Gerchberg-Saxton (GSW) approach to generate controllable, aberration-free dual-focus beams [91]. These beams form double-layer cracks within the SiC, achieving high-efficiency, low-loss, simultaneous double-layer slicing of SiC wafers, with a slicing speed twice as fast as conventional techniques. The team successfully sliced a 10 × 10 × 1.4 mm^3^ SiC sample into three wafers with thicknesses ranging from 400 to 550 μm. The tensile strengths required to separate the two layers were 1.07 MPa and 1.40 MPa, and the resulting wafers achieved minimum surface roughness values of 0.9 μm and 0.55 μm, respectively.

Despite the significant application potential of scanning galvanometer and multi-focus parallel processing technologies, their technical maturity requires further improvement, and both encounter distinct challenges in achieving large-scale industrial application. For scanning galvanometer technology, precisely controlling field distortion and focal consistency across large areas remains difficult. Furthermore, dynamic errors during high-speed scanning and inherent thermal drift limit the long-term stability essential for high-precision processing. Similarly, the design and calibration of DOE, critical to multi-focus parallel processing, are considerably complex, with ensuring high energy uniformity among the foci posing a significant challenge. Therefore, continued optimization in areas such as optical system design, dynamic error compensation, and processing flexibility is crucial to fully leverage the advantages of these technologies.

#### Stripping process optimization

In the production of high-quality SiC wafers, the peeling process, as a core step in wafer processing, plays a decisive role in the final product’s quality, precision, and production efficiency. Therefore, in-depth research and process optimization of the peeling technique are crucial. Currently, the commonly used mechanical peeling methods have certain limitations [37, 42, 74, 78]. First, during the mechanical peeling process, microcracks or fractures are easily generated in the wafer, which not only affects the yield but also may negatively impact subsequent processing stages. Second, mechanical peeling relies on bonding/debonding techniques, which are inefficient and complex, making it difficult to meet the demands of large-scale production. Additionally, the mechanical stress applied to the wafer during peeling may cause surface damage, further degrading product quality. This indicates that the exploration of more efficient and controllable peeling technologies has become a pressing issue in the SiC wafer manufacturing field.

**Fig. 11:**
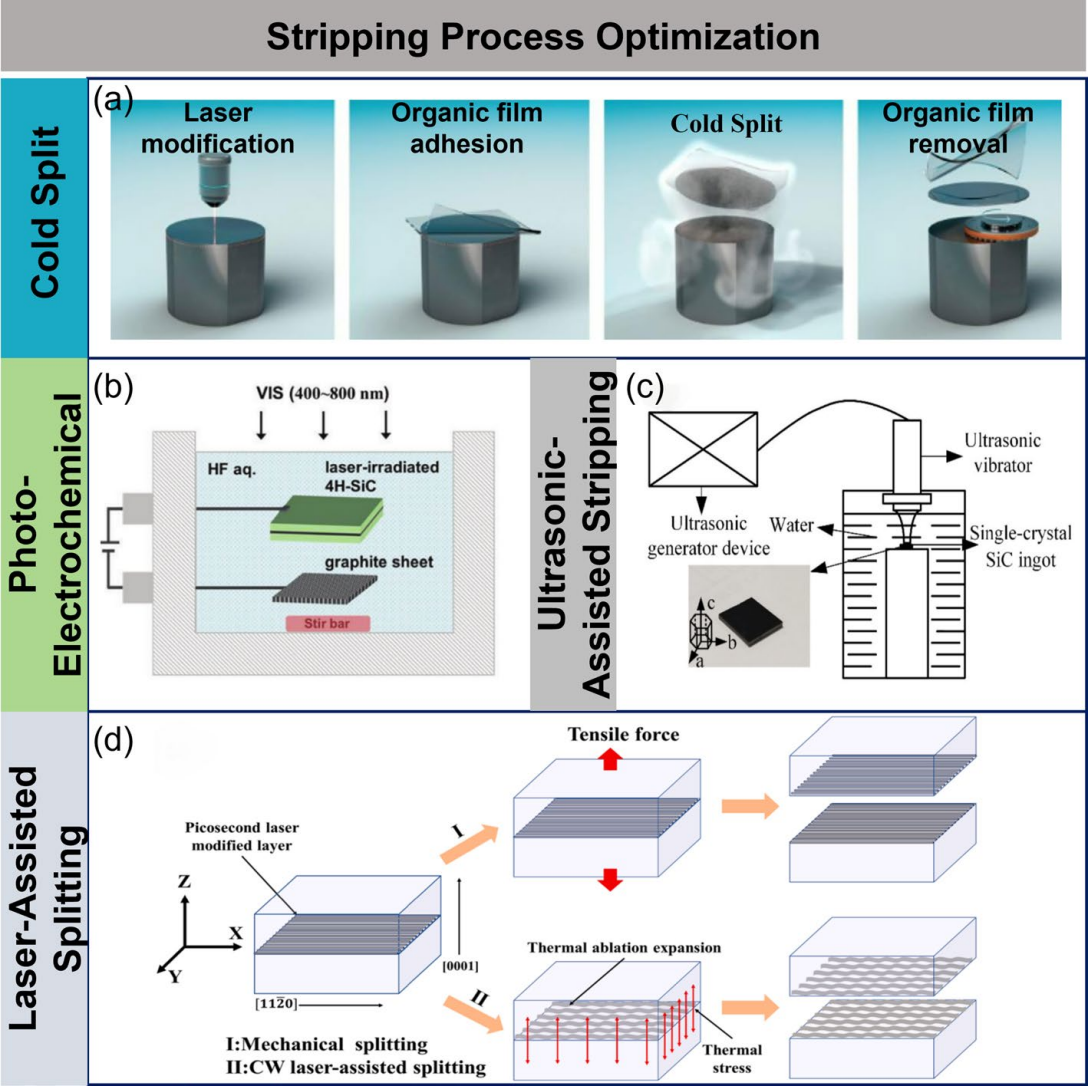
Stripping process optimization. **a** Cold split [92] **b** Photo-Electrochemical [93] **c** Ultrasonic-assisted stripping [94] **d** Laser-assisted splitting [98]

Infineon’s COLD SPLIT technology is currently the most advanced wafer separation technology in the world [92]. This process uses pulse lasers to process SiC wafers, forming a horizontal modified layer (Fig. 11a). Then, a sacrificial layer and polymer film are applied, and rapid cooling induces shrinkage of the polymer, generating stress for lateral separation. This technology significantly reduces material loss, improves wafer TTV and surface roughness, and can increase SiC wafer production capacity by nearly three times, making it especially suitable for large-diameter wafer separation. To further break through technical bottlenecks, researchers are actively exploring other novel separation technologies, (Fig. 11b). Geng et al. developed a bandgap-selective photoelectrochemical (PEC) etching technology, which uses hydrofluoric acid solution during the PEC process to achieve selective oxidation and corrosion of the modified layer, avoiding damage to the unmodified layer and providing an innovative low-loss separation solution for SiC wafers [93].

However, this technology faces challenges such as environmental risks from highly corrosive chemicals and longer etching times that affect production efficiency. To address these challenges, Lv et al. proposed an ultrasonic vibration-assisted separation technology, which effectively promotes the expansion of internal cracks in SiC, reducing the separation force by 25–60% [94]. Studies have shown that under optimized conditions, the separation force can be reduced by up to 60%, and wafer separation can be directly achieved with appropriate ultrasonic power and vibration time (Fig. 11c).

Despite some progress, SiC wafer separation technology still requires continuous improvements in quality, precision, and efficiency. The development of novel multi-laser composite-assisted crack induction and propagation separation technology has become a key area of research. Lumley’s proposed laser-induced thermal stress controllable fracture technique offers a new direction for non-contact processing, showing advantages in controlling prefabricated crack trajectories, and has already been applied in several fields [95]. Typical applications of this technology include: Huang et al. used a 355 nm Nd: YAG laser to generate modified layers and induce crack propagation within glass, while Deng et al. achieved precise cutting of 12 mm thick KDP crystals using femtosecond lasers and created thermal stress near cracks using continuous lasers to achieve efficient separation [96, 97]. Building on these technological advances, Jiang et al. successfully applied laser-assisted separation technology to SiC wafers, proposing a continuous-wave laser-assisted separation method based on a picosecond laser-induced modified layer [98] (Fig. 11d). They optimized the laser parameters and found that the amorphous carbon in the modified area significantly improved the sample’s laser absorption, with the absorption coefficient increasing by 2720 times at 1064 nm, generating enough thermal stress to achieve precise separation. This breakthrough provides a new solution for SiC wafer separation.

Table 1 summarizes the research progress in SiC ingot modification and separation technologies by various organizations, all with the core goal of achieving small modification layers, high efficiency, and low loss. The results show that through material pre-treatment, laser parameter optimization (such as wavelength, pulse duration, etc.), and the synergistic effect of auxiliary separation processes, significant improvements in processing performance have been achieved. Currently, the peeling layer thickness has been optimized to about 120 μm, with total material loss controlled between 30% and 50%. However, balancing processing quality and efficiency remains challenging, and further in-depth research is urgently needed to achieve technological breakthroughs and scalable applications for cost reduction and efficiency improvement in SiC crystal separation.

**Table 1:** Process solutions and key technical indicators achieved by various research units in SiC laser modification and stripping technology

Unit	Modification	Stripping	Quality			Efficiency	Cost per wafer
			Modified layer thickness	Material loss	Other parameters		
INFINEON	fs Dual-pulse mode	Cold Split	< 200 μm	< 80 μm	–	6-inch 10–30 min	20 mm Ingot→ 80 wafers
DISCO	fs, KABRA Dual-pulse mode	Mechanical and ultrasonic	350 μm	~ 100 μm	TTV: 5 μm Warp: 1.6 μm	6-inch 17 min	–
HALO	–	–	–	–	–	6-inch With a projected annual production capacity of 12,000 wafers	–
CETC	fs(1030 nm) Single-pulse mode	Thermal split	350–500 μm	~ 180 μm	Sa: ≤20 μm Bow: ≤±20 μm	6-inch ≤ 15 min	–
JINGFEI	fs(1030 nm)	Mechanical stripping	-	100–160 μm	–	–	20 mm Ingot→ 45 wafers
GIE	–	–	~ 130 μm	–	TTV: ≤5 μm Warp: ≤20 μm Bow: ≤10 μm Yield: 95%	6-inch 10 min 8-inch 17 min With a projected annual production capacity of 20,000 wafers	–
WESTLAKE	-	Suction cup stripping	100–120 μm	Semi-Insulating Type<60 μm Conductive Type<120 μm Total Material Loss Rate 30–50%	-	6-inch ≤ 30 min	–
HAN’S LASER	Quality crystal bonding (1039 nm)	Basal slip	175 μm 350 μm	~ 100 μm	Yield:99.5%	400–1000 mm/s Precision: ±1 μm	–
DELPHILASER	–	–	–	100 μm	–	2.5 min/inch	–
CAS	ps(1064 nm) Single-pulse mode	Laser stripping	–	–	Sa:1.8 μm	Repeat etching Process Efficiency:60%	Semi-Insulating and conductive-type 4H-SiC Samples
	ps(1045 nm) Multi-focus parallel	Mechanical stripping	–	–	Sa:432 nm	More efficient than single-focus	
	ps(1064 nm) Multi-focus parallel	Mechanical stripping	123.68∼171.6 μm	–	Sa:0.55–1.432 μm	More efficient than single-focus	
ZheJiang University	fs(1030 nm)	Photo-electrochemical	–	~ 20 μm	Sa:1 μm	Average etching rate:0.33 cm^2^/h	
ZhongBei University	fs(520 nm) Single-pulse mode ps(1030 nm) Single-pulse mode	Ultrasonic vibration	500 μm	–	Sa:6 μm	Modification:≤5 min Ultrasonic vibration:5 min	
XiaMen University	ns(1064 nm) Scanning Galvanometer	Laser stripping	250 μm	915 nm	Sa:168 μm	Laser 1: 18–75 mm/s Laser 2: 2000–3000 mm/s	

TTV, Total thickness variation; CETC, The 2ND Research Institute of China Electronics Technology Group Corporation; JINGFEI, Jingfei Semiconductor; GIE, Jiangsu General Science Technology Co.,Ltd; WESTLAKE, Westlake Instruments; HAN’S LASER, Han’s Laser Technology Industry Group Co., Ltd; CAS, Institute of Semiconductors, Chinese Academy of Sciences; Sa, Surface roughness

## Summary and outlook

This paper systematically investigates the interaction mechanism between lasers and wide-bandgap semiconductor materials, providing an in-depth analysis of the critical impact of laser pulse characteristics (such as pulse width and energy intensity) and material electronic states on energy transfer. By comparing the nonlinear ionization process of ultra-short pulse lasers with the thermal effects of long pulse lasers, it reveals significant differences in energy deposition under various laser parameters. By integrating the electronic state distribution of transparent brittle materials, this study elucidates the dynamic evolution of laser energy transfer and material modification structures, revealing the complex mechanisms underlying laser-material interactions. These findings establish a theoretical foundation for the laser processing of wide-bandgap semiconductors. Furthermore, this paper systematically summarizes and analyzes the key issues and modification mechanisms in the laser-induced SiC modification and stripping process. It provides an in-depth multi-scale analysis, ranging from the macroscopic formation of ablation craters, mesoscopic chemical bond breakage, to microscopic phase transformations, offering scientific guidance for the optimization of laser processing techniques. In terms of process optimization, this paper provides a detailed discussion on the comprehensive optimization pathway, ranging from material pretreatment (crystal orientation calibration and surface roughness measurement) to the quality enhancement of the modified layer (involving pulse width, scanning speed, multi-pulse sequences, and parameter coordination). It systematically summarizes strategies for efficiency improvement and process refinement of the stripping technique, offering a systematic solution for the efficient and high-quality laser processing of SiC wafers.

Looking ahead, laser slicing technology, as a multidisciplinary technology integrating optics, materials, thermal science, and mechanics, demonstrates excellent material adaptability and capabilities for large-scale wafer processing. However, this technology still requires further iteration and advancement. On one hand, there is a need to transition from traditional single-focus modification modes to multi-focus parallel modification to enhance the efficiency of large-scale wafer processing. On the other hand, the development of precise temperature field modeling and residual stress control techniques holds potential to significantly improve the utilization rate of ingots. Additionally, addressing issues such as uneven light absorption caused by crystal growth defects that affect slicing quality, future efforts should focus on developing intelligent optimization systems based on online detection technology to achieve real-time and precise control of the laser modification process. It is foreseeable that laser slicing technology will provide robust technical support for advancements in semiconductors, optoelectronics, and related fields.

## Data Availability

No datasets were generated or analysed during the current study.
